# Molecular response to aromatase inhibitor treatment in primary breast cancer

**DOI:** 10.1186/bcr1732

**Published:** 2007-06-07

**Authors:** Alan Mackay, Ander Urruticoechea, J Michael Dixon, Tim Dexter, Kerry Fenwick, Alan Ashworth, Suzanne Drury, Alexey Larionov, Oliver Young, Sharon White, William R Miller, Dean B Evans, Mitch Dowsett

**Affiliations:** 1 The Breakthrough Breast Cancer Research Centre, The Institute of Cancer Research, Fulham Road, London, SW3 6JB, UK; 2 Academic Department of Biochemistry, Royal Marsden Hospital, Fulham Road, London, SW3 6JJ, UK; 3 The Edinburgh Breast Unit, Western General Hospital, Edinburgh, EH4 2XU, UK; 4 Novartis Pharma AG, Basel, Switzerland

## Abstract

**Background:**

Aromatase inhibitors such as anastrozole and letrozole are highly effective suppressants of estrogen synthesis in postmenopausal women and are the most effective endocrine treatments for hormone receptor positive breast cancer in such women. Little is known of the molecular effects of these agents on human breast carcinomas *in vivo*.

**Methods:**

We randomly assigned primary estrogen receptor positive breast cancer patients to treatment with anastrozole or letrozole for 2 weeks before surgery. Expression profiling using cDNA arrays was conducted on pretreatment and post-treatment biopsies. Sample pairs from 34 patients provided sufficient RNA for analysis.

**Results:**

Profound changes in gene expression were seen with both aromatase inhibitors, including many classical estrogen-dependent genes such as *TFF1*, *CCND1*, *PDZK1 *and *AGR2*, but also many other genes that are likely to represent secondary responses; decrease in the expression of proliferation-related genes were particularly prominent. Many upregulated genes are involved in extracellular matrix remodelling, including collagens and members of the small leucine-rich proteoglycan family (*LUM*, *DCN*, and *ASPN*). No significant differences were seen between letrozole and anastrozole in terms of molecular effects. The gene changes were integrated into a Global Index of Dependence on Estrogen (GIDE), which enumerates the genes changing by at least twofold with therapy. The GIDE varied markedly between tumours and related significantly to pretreatment levels of HER2 and changes in immunohistochemically detected Ki67.

**Conclusion:**

Our findings identify the transcriptional signatures associated with aromatase inhibitor treatment of primary breast tumours. Larger datasets using this approach should enable identification of estrogen-dependent molecular changes, which are the determinants of benefit or resistance to endocrine therapy.

## Introduction

Approaching 80% of human breast carcinomas express estrogen receptor (ER)-α protein at clinically significant levels and are considered ER positive. Estrogen deprivation, or antagonism, is an effective treatment for many but not all patients with such tumours. The selective ER modifier tamoxifen has been the predominant treatment for the past two decades and improves survival in ER-positive patients receiving this as adjuvant therapy after surgery [1]. However, in postmenopausal women aromatase inhibition with the nonsteroidal inhibitors anastrozole and letrozole has now been shown to be more effective than tamoxifen as adjuvant therapy [2]. Letrozole and anastrozole are highly specific for the aromatase enzyme and inhibit whole body aromatization by 99% and 97%, respectively [3]. Aromatase inhibitors (AIs) therefore confer highly selective and essentially complete withdrawal of estrogen in postmenopausal patients.

Proliferation of malignant cells, as measured by expression of the nuclear antigen Ki67, is reduced in more than 90% of ER-positive primary breast carcinomas by treatment with AIs [4,5]. This suggests that almost all ER-positive tumours derive some proliferative stimulus from estrogen and may be considered hormone responsive; in some patients, however, this effect may be only modest. We recently found that the difference in the change in Ki67 after 2 weeks of treatment with anastrozole or tamoxifen, or the two drugs in combination was predictive of relative recurrence-free survival in a parallel adjuvant trial of the same treatments [6]. Additionally, Ki67 levels after 2 weeks of treatment significantly correlated with recurrence-free survival in the same patients in the presurgical study [7]. Both of these findings support the validity of short-term changes in Ki67 as an intermediate marker of the clinical effectiveness of endocrine therapy. It seems likely, however, that Ki67 is an imperfect marker of proliferation and that changes in gene expression other than those related to proliferation may be involved in determining the clinical effectiveness of estrogen deprivation.

Transcriptional profiling of estrogen responses in ER-positive human breast cancer cell lines and model systems *in vitro *leads to changes in the transcription of large numbers of genes [8,9], but very little is known of these effects *in vivo *or how these effects vary between tumours and whether these molecular changes fully encompass the determinants of clinical response. Biopsy of tumours before and during presurgical treatment with an AI allows the study of estrogen-dependent effects across a range of ER-positive breast carcinomas *in situ*.

We therefore evaluated the effects of estrogen deprivation with letrozole or anastrozole on Ki67 expression and transcriptional profiles in ER-positive breast carcinomas *in vivo*. Such an approach might provide insights into the mechanisms of clinical benefit and allow the development of a predictor of that benefit.

Our specific aims in the present study were as follows: to determine whether there is a significant difference between letrozole and anastrozole in terms of change in Ki67 (reported elsewhere) and changes in gene transcription; to identify the genes that change with aromatase inhibition and to integrate these as a Global Index of Dependence on Estrogen (GIDE); to assess how the most prominent gene changes relate to those reported *in vitro *with estrogen stimulation; and to determine the relationship between the GIDE and previously described putative determinants of benefit from endocrine therapy such as HER2 and Ki67 expression.

## Materials and methods

### Patient samples

Postmenopausal patients with primary ER-positive (Allred scores 2 to 8; note that scores of 2 are conventionally regarded as ER negative [10]) breast cancer were randomly assigned to presurgical treatment for 2 weeks with letrozole (2.5 mg/day orally) or anastrozole (1 mg/day orally). Multiple core-cut biopsies were taken with a 14-guage needle before treatment and at surgery from 54 patients, and were either immediately frozen in liquid nitrogen for RNA analysis or were fixed in neutral buffered formalin for immunohistochemistry. RNA from each frozen biopsy was extracted using Trizol (Invitrogen, Paisley, UK), in accordance with the manufacturer's instructions.

### Microarray hybridizations

Total RNA integrity was confirmed on an Agilent 2100 BioAnalyser (Agilent Technologies, South Queensferry, West Lothian, UK) before linear T7 amplification using a RiboAmp kit (Arcturus/Molecular Devices Corporation Sunnyvcale, CA, USA). Amplified aRNA (4 μg) was labeled with either cy3 or cy5 and hybridized to Breakthrough 17K cDNA microarrays in replicate dye swap hybridizations, as previously described [11]. The Breakthrough 17K microarray platform and all primary microarray data have been submitted to Array Express (ArrayExpress submission number E-TABM-180). Annotation of the Breakthrough 17K cDNA microarray based on build 189 of Unigene is provided as Additional file 6.

### Analysis of microarray data

Expression values from spots with hybridization artefacts or extremely low intensities were flagged in Genepix 5.1 (Axon Instruments/Molecular Devices Corporation Sunnyvcale, CA, USA) and then converted to missing values and removed from the analysis. The raw intensity values were then converted to log_2 _ratios of sample to reference (M values) and log_2 _average spot intensity (A values) for all subsequent pre-processing and analysis. The loess local regression function was used to remove biases resulting from the combined effect of spot intensity and the row group to which the spot belonged, and then to remove the more global bias across the slide. A quantile filter was used to remove data that had average intensity or A values below the 25th percentile in 60% or more of the hybridizations. The M values for each hybridization were rescaled so as to remove the relationship between increasing dispersion of M values with increasing dispersion of A values across the hybridisations. This latter transformation did not involve extensive rescaling of the data and although it clarified the relationships found in this study, these were all apparent without this step. The replicate dye swap hybridizations were then averaged. This left 14,024 genes that were used for paired (pre/post) differential gene expression analysis using SAM version 2.21 [12]. In order to focus on the more extensive gene fluctuations between samples, further reductions in the number of genes used for some analyses were based on filtering out genes with low inter-sample variation. We used the interquartile range as a robust estimate of gene variation and used a stringent threshold at interquartile range = 0.75 (2,418 genes remaining).

In order to map the gross phenotypic changes across the samples, the following supervised analysis was chosen. A core set of genes was selected using the weighted Kolmogorov-Smirnov statistic because of its robustness and flexibility. The maximum number of genes (421) that gave minimum leave-one-out cross-validation in separating pretreatment from post-treatment samples using the k-nearest-neighbor algorithm (k = 7) was identified, and these genes were retained. Agglomerative clustering (see below) was used to separate the 421 selected genes into 10 clusters of co-regulated genes. Each cluster was then represented by the average M value of its genes for each sample (metagene) following centering and rescaling across samples. All clustering used the flexible β agglomerative clustering algorithm with a correlation distance measure for both genes and arrays. Clustering heat maps were produced with Java Treeview 1.0.12 software. Correlations were performed with Spearman rank or Pearson correlations.

### Immunohistochemistry

Conventional immunohistochemistry was performed on each biopsy using antibodies for ER-α clone 6F11 (Novocatsra Laboratories, Newcastle. upon Tyne, UK), progesterone receptor (PgR) clone PgR 636 (Dako Ltd., Ely, Cambridgeshire, UK), and KI67 clone MIB-1 (DAKO), in accordance with the manufacturers' instructions.

ER and PgR immunohistochemistry were quantitated according to Allred score [10]. Ki67 immunohistochemistry is reported as the number of positive cells among 1,000 malignant cells counted and is expressed as a percentage.

### Real-time PCR

Quantitative real-time PCR was conducted in five genes of interest (*CCND1*, *PDZK1*, *FAS*, *TFF1*, and *MAN1A1*). Total RNA from the same RNA preparations as used for microarray analysis was reverse transcribed using random primers and Superscript III (Invitrogen), in accordance with the manufacturer's instructions. A reverse transcription negative control was included to account for any genomic DNA contamination. cDNA samples were subjected to quantitative PCR using Taqman^® ^(Applied Biosystems, Warrington, UK) on an ABI Prism 7900 HT with primers designed by Primer Express, or Quantitect SYBR green (Qiagen, Crawley, West Sussex, UK) on an Opticon Monitor 2 with primers designed by Primer 3 in two different laboratories. Primer sequences are shown in Additional file 1. Pretreatment/post-treatment changes were estimated after normalization using the geometric mean of the two reference genes that had been shown to be unchanged in expression during treatment with AIs (*TBP *and *KIAA0674*).

## Results

RNA of sufficient quality and quantity was obtained from 34 pretreatment/post-treatment pairs of samples. The following findings refer solely to those samples. Patient clinical information is summarized in Additional file 2.

The samples were clustered to determine whether pretreatment and post-treatment biopsies aggregated together as nearest neighbors in clustering dendrograms. Half of pre/post biopsy pairs were found to co-aggregate whether based on all 14,034 measured genes (17/34) or the 2,418 genes remaining following filtering to retain the most variable genes (18/34). Similar proportions of co-aggregating pairs were also identified using other algorithms (for instance, complete linkage and group average linkage; data not shown). Separation of paired biopsies in this analysis contrasts with other studies in which the differences in gene expression among breast tumours is far greater than that observed as a result of treatment with chemotherapeutic agents [11,13].

**Figure 1:**
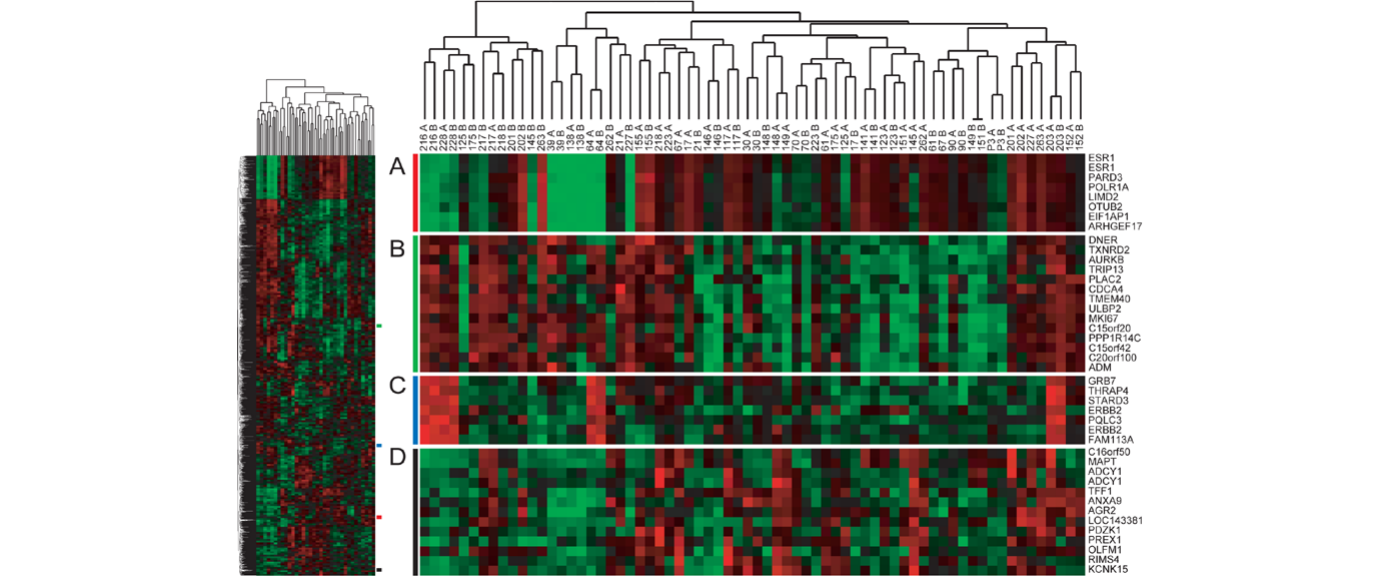
Heatmap of unsupervised clustering of pretreatment and post-treatment biopsies. A heatmap of the unsupervised clustering of the 34 pretreatment and post-treatment samples (labeled A and B respectively) using 2,418 of the most variable genes is shown. The entire heatmap is shown in miniature on the left. Clusters containing the genes **(a) ***ESR1*, **(b) ***MKI67*, **(c) ***ERBB2 *and **(d) ***TFF1 *are shown in detail. Out of 34 pairs of biopsies, 18 co-aggregated at the first or second level in the sample dendrogram.

A heat map diagram from clustering of the 2,418 most variable genes among the 68 biopsies is shown in Figure 1. Clusters of genes containing some of the most important known markers of breast tumour phenotype are shown in greater detail: *ESR1 *(Figure 1a), *MKI67 *(Figure 1b), *ERBB2 *(Figure 1c), and *TFF1 *(Figure 1d). Levels of *ESR1 *and *ERBB2 *gene expression were inversely correlated in these samples (*r *= -0.57, *P *= 0.0005, Pearson correlation), as has been shown in many other studies of breast tumours. The samples with the lowest *ESR1 *or high *ERBB2 *invariably had pre/post biopsy pairs that co-aggregated as nearest neighbours and accounted for more than half (11/17) of the co-aggregating pairs. The *ERBB2 *cluster contained several genes that are present in the *ERBB2 *amplicon on chromosome 17q12-21, including *GRB7*, *THRAP4*, and *STARD3*, and were highly over-expressed in the four HER2 amplified cases. Data files for Java Treeview are provided as supplementary information (Additional files 7, 8, 9, 10).

**Figure 2:**
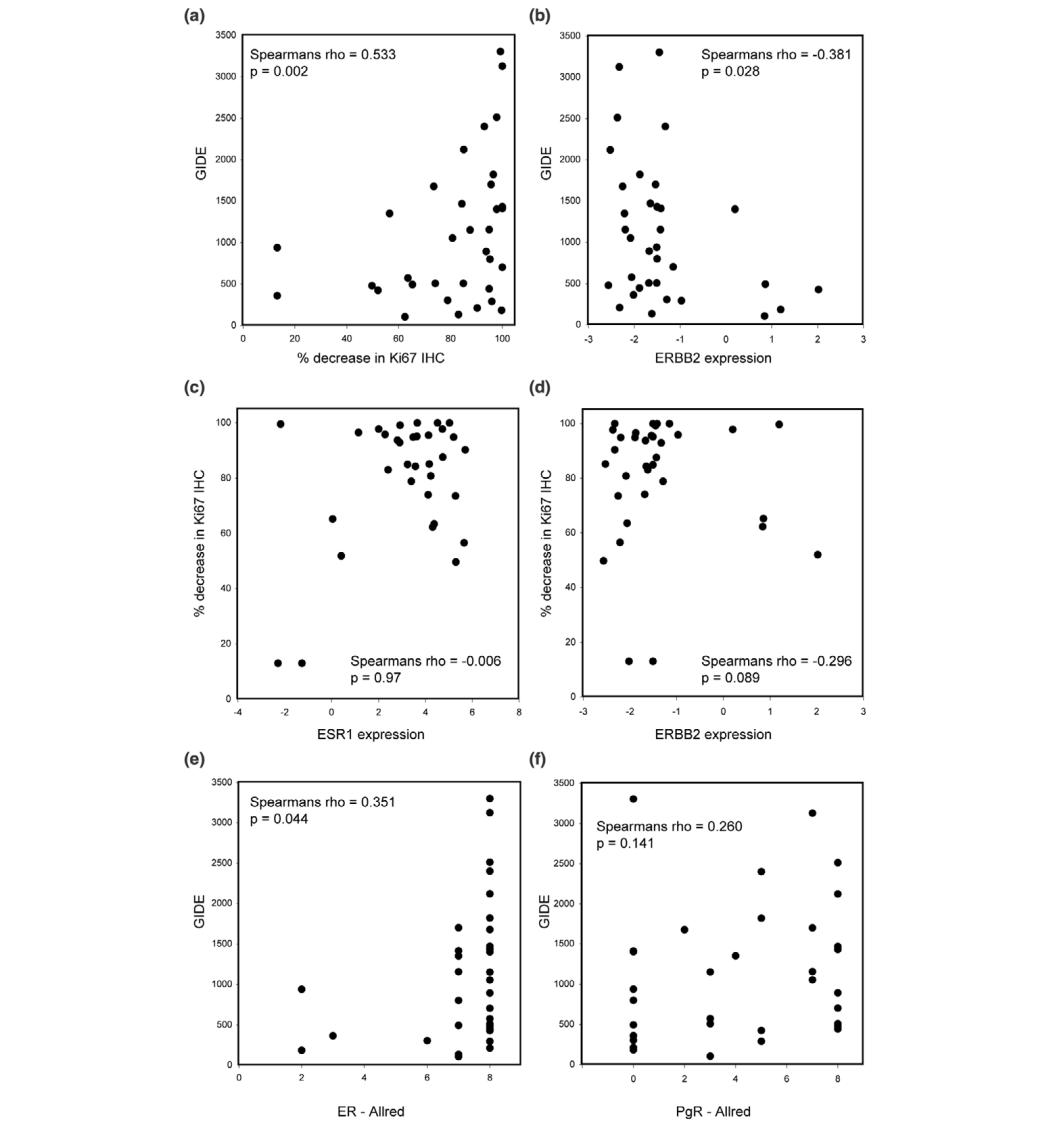
Comparison of GIDE score and change in Ki67 immunohistochemistry with *ESR1 *and *ERBB2 *expression. **(a) **Significant positive correlation of the Global Index of Dependence on Estrogen (GIDE) scores for each pair of biopsies with percentage decrease in Ki67 immunohistochemistry (IHC) is shown. **(b) **Significant negative correlation of the GIDE score to the pretreatment expression of *ERBB2*, as derived from microarray profiling, is shown. Also shown are comparisons of the change in Ki67 immunohistochemistry (% decrease) is shown with pretreatment **(c) ***ESR1 *expression and **(d) ***ERBB2 *expression. Finally, comparisons of GIDE scores with pretreatment immunohistochemical measurements (Allred scores) are shown for **(e) **estrogen receptor (ER) and **(f) **progesterone receptor (PgR).

To summarize the effects of estrogen deprivation on gene expression, we have derived a Global Index of Dependence on Estrogen (GIDE). This index was defined as the number of genes changing by at least twofold between each pair of biopsies irrespective of the direction of change. This index correlated positively with change in the proliferation marker Ki67 (Spearman rank rho = 0.533, *P *= 0.0022; Figure 2a) and negatively with the expression of *ERBB2 *(Spearman rank rho = -0.381, *P *= 0.0282; Figure 2b). Although no patients with a high GIDE were among the lowest in terms of *ESR1 *expression, overall there was not a significant correlation between the two. A complete summary of GIDE data is provided in Additional file 3.

The primary end-point of the study was the reduction in tumour proliferation measured as the change in the biomarker Ki67 by conventional immunohistochemistry. The relationship between change in Ki67 immunohistochemistry and microarray expression of *ESR1 *and *ERBB2 *is shown in Figure 2c,d. Tumours expressing low levels of ER or high levels of *ERBB2 *exhibited less reduction in Ki67 staining following AI treatment.

Correlations of the GIDE with immunohistochemical measurements of ER and PgR (Allred score) are shown in Figure 2e,f. In these samples there was a significant correlation of GIDE with pretreatment ER staining but not with that of PgR. There was no significant difference between letrozole and anastrozole in their effects on the GIDE or on Ki67, confirming the result for the whole patient set [14].

A paired SAM statistical analysis identified 1,395 genes that were upregulated and 1,264 genes that were downregulated by AI treatment using a local false discovery rate threshold of 1%. Significantly changing genes were then ranked according to their average fold change; the top 40 downregulated genes are listed in Table 1 and the top 40 upregulated genes are listed in Table 2 (the complete list is shown in Additional file 4). The most consistently downregulated genes included *TFF1*, *PDZK1*, *AGR2*, *TFF3*, *STC2*, and *CCND1*. The most consistently upregulated genes included *LUM*, *CALD1*, *ASPN*, *DCN*, *PDGFRA*, *VIM*, *SPARC*, *MAN1A1*, and *FAS*. Quantitative real-time PCR confirmed significant upregulation of *MAN1A1 *and *FAS *(*P *< 0.05 for each) and downregulation of *TFF1*, *PDZK1*, and *CCND1 *(*P *< 0.01, *P *< 0.001 and *P *< 0.001, respectively; data not shown). The complete list of upregulated and downregulated genes was subjected to Gene Ontology analysis using Onto-Express and Pathway Express [15].

**Table 1:** Genes downregulated by AI treatment

ID	Symbol	Description	Unigene	Fold	LFDR
HSI182A05	*TFF1*	Trefoil factor 1 (breast cancer, estrogen-inducible)	Hs.162807	0.26	0.09
HSI054D07	*HBB*	Haemoglobin, beta	Hs.523443	0.31	0.00
HSI047B01	*PDZK1*	PDZ domain containing 1	Hs.444751	0.35	0.00
HSI035H02	*CYP2B6*	Cytochrome P450, family 2B6	Hs.1360	0.41	0.04
HSI075H09	*AGR2*	Anterior gradient 2 homolog (*Xenopus laevis*)	Hs.530009	0.41	0.08
HSI031E07	*STARD10*	START domain containing 10	Hs.188606	0.42	0.00
HSI183G10	*TFF3*	Trefoil factor 3 (intestinal)	Hs.82961	0.43	0.00
HSI147F09	*ZBTB20*	Zinc finger and BTB domain containing 20	Hs.570657	0.45	0.00
HSI182A08	*STC2*	Stanniocalcin 2	Hs.233160	0.47	0.00
HSI147F08	*KTN1*	Kinectin 1 (kinesin receptor)	Hs.509414	0.47	0.00
HSI059H10	*LOC143381*	Hypothetical protein LOC143381	Hs.388347	0.47	0.00
HSI147F10	*MSI2*	Musashi homolog 2 (*Drosophila*)	Hs.134470	0.49	0.00
HSI177G07	*EST*	Transcribed locus	Hs.443277	0.50	0.10
HSI053H02	*UBE2C*	Ubiquitin-conjugating enzyme E2C	Hs.93002	0.52	0.10
HSI096C06	*MAPT*	Microtubule-associated protein tau	Hs.101174	0.52	0.00
HSI049A02	*ERGIC1*	ER-golgi intermediate compartment 1	Hs.509163	0.53	0.09
HSI040C08	*AZGP1*	Alpha-2-glycoprotein 1, zinc	Hs.546239	0.55	0.00
HSI133F06	*EST*	Transcribed locus	Hs.159264	0.55	0.00
HSI182E08	*PLAT*	Plasminogen activator, tissue	Hs.491582	0.55	0.00
HSI033B05	*LY6E*	Lymphocyte antigen 6 complex, locus E	Hs.521903	0.56	0.00
HSI048F12	*CCND1*	Cyclin D_1_	Hs.523852	0.56	0.10
HSI085G12	*KCNK15*	Potassium channel, subfamily K, member 15	Hs.528664	0.57	0.00
HSI177H07	*PCBP3*	Poly(rC) binding protein 3	Hs.474049	0.57	0.10
HSI032D02	*ABCA3*	ATP-binding cassette, subfamily A3 (ABC1)	Hs.26630	0.57	0.00
HSI182A02	*TFF3*	Trefoil factor 3 (intestinal)	Hs.82961	0.58	0.04
HSI025A03	*FGD3*	FYVE, RhoGEF and PH domain containing 3	Hs.411081	0.58	0.00
HSI070B06	*AP1S1*	Adaptor-related protein complex 1, sigma 1 subunit	Hs.489365	0.58	0.08
HSI057H12	*GNB2*	Guanine nucleotide binding protein beta 2	Hs.185172	0.58	0.00
HSI080H11	*SEMA3F*	Semaphorin 3F	Hs.32981	0.59	0.00
HSI054G06	*NUSAP1*	Nucleolar and spindle associated protein 1	Hs.511093	0.59	0.00
HSI124D07	*RIMS4*	Regulating synaptic membrane exocytosis 4	Hs.517065	0.59	0.00
HSI065C09	*CNNM2*	Cyclin M2	Hs.500903	0.59	0.00
HSI080F02	*PREX1*	PIP3-dependent RAC exchanger 1	Hs.153310	0.59	0.00
HSI095H09	*C6orf97*	Chromosome 6 open reading frame 97	Hs.130239	0.59	0.00
HSI161G02	*EST*	Transcribed locus	Hs.570637	0.60	0.00
HSI045G02	*UBE2T*	Ubiquitin-conjugating enzyme E2T (putative)	Hs.5199	0.60	0.00
HSI046F10	*TOP2A*	Topoisomerase (DNA) II alpha 170 kDa	Hs.156346	0.60	0.00
HSI183D04	*AR*	Androgen receptor	Hs.496240	0.61	0.01
HSI183A08	*SLC9A3R1*	Solute carrier family 9, member 3 regulator 1	Hs.396783	0.61	0.00
HSI025G02	*SHARPIN*	SHANK-associated RH domain interactor	Hs.529755	0.61	0.08

Shown are the first 40 downregulated genes from a paired SAM analysis, which identified 1,264 genes to be downregulated by aromatase inhibitor (AI) treatment below a local false discovery rate of 1% (LFDR). The genes are ranked according to their fold change.

**Table 2:** Genes upregulated by AI treatment

ID	Symbol	Description	Unigene	Fold	LFDR
HSI022G08	*LUM*	Lumican	Hs.406475	2.87	0.11
HSI101E05	*ODF2L*	Outer dense fiber of sperm tails 2-like	Hs.149360	2.80	0.07
HSI027H04	*IGJ*	Immunoglobulin J polypeptide	Hs.381568	2.73	0.07
HSI082D05	*RNH1*	Ribonuclease/angiogenin inhibitor 1	Hs.530687	2.51	0.00
HSI056B04	*COL3A1*	Collagen, type III, alpha 1	Hs.443625	2.50	0.00
HSI182D05	*MRC1L1*	Mannose receptor, C type 1	Hs.461247	2.45	0.06
HSI067F08	*C21orf70*	Chromosome 21 open reading frame 70	Hs.410830	2.44	0.00
HSI127E04	*CALD1*	Caldesmon 1	Hs.490203	2.37	0.11
HSI030C06	*PTPRC*	Protein tyrosine phosphatase, receptor type, C	Hs.192039	2.36	0.11
HSI067H02	*ASPN*	Asporin (LRR class 1)	Hs.435655	2.34	0.00
HSI066B08	*COL14A1*	Collagen, type XIV, alpha 1 (undulin)	Hs.409662	2.28	0.11
HSI049B12	*COL1A2*	Collagen, type I, alpha 2	Hs.489142	2.28	0.00
HSI049G07	*DCN*	Decorin	Hs.156316	2.28	0.00
HSI067E05	*MRC1L1*	Mannose receptor, C type 1	Hs.461247	2.22	0.05
HSI101D05	*IFT122*	Intraflagellar transport 122 homolog (Chlamydomonas)	Hs.477537	2.17	0.03
HSI183E05	*PDGFRA*	Platelet-derived growth factor receptor, alpha	Hs.74615	2.14	0.09
HSI031A12	*FSTL1*	Follistatin-like 1	Hs.269512	2.12	0.00
HSI183H01	*COL5A2*	Collagen, type V, alpha 2	Hs.445827	2.11	0.00
HSI055A11	*ECM2*	Extracellular matrix protein 2	Hs.117060	2.08	0.09
HSI018G02	*SPON1*	Spondin 1, extracellular matrix protein	Hs.445818	2.06	0.00
HSI183B01	*PDGFRA*	Platelet-derived growth factor receptor, alpha	Hs.74615	2.04	0.01
HSI037C02	*CPVL*	Carboxypeptidase, vitellogenic-like	Hs.233389	2.03	0.06
HSI062B08	*SAS10*	Disrupter of silencing 10	Hs.322901	2.01	0.00
HSI129E10	*ADAM12*	ADAM metallopeptidase domain 12 (meltrin alpha)	Hs.386283	2.00	0.00
HSI183G08	*RGS1*	Regulator of G-protein signalling 1	Hs.75256	1.98	0.18
HSI054F01	*VIM*	Vimentin	Hs.533317	1.97	0.00
HSI048C08	*CTGF*	Connective tissue growth factor	Hs.410037	1.97	0.00
HSI183G05	*SPARC*	Secreted protein, acidic, cysteine-rich (osteonectin)	Hs.111779	1.96	0.00
HSI139G09	*ADAMTS2*	ADAM metallopeptidase with thrombospondin motif 2	Hs.23871	1.96	0.00
HSI018D05	*FBLN1*	Fibulin 1	Hs.24601	1.95	0.00
HSI040E08	*DUSP1*	Dual specificity phosphatase 1	Hs.171695	1.95	0.00
HSI082C05	*MAN1A1*	Mannosidase, alpha, class 1A, member 1	Hs.102788	1.94	0.01
HSI098G12	*RARRES1*	Retinoic acid receptor responder 1	Hs.131269	1.94	0.00
HSI044C09	*SAT*	Spermidine/spermine N1-acetyltransferase	Hs.28491	1.92	0.09
HSI030F05	*HTRA1*	HtrA serine peptidase 1	Hs.501280	1.92	0.00
HSI088D11	*CILP*	Cartilage intermediate layer protein	Hs.442180	1.91	0.00
HSI040E09	*MME*	Membrane metallo-endopeptidase (CALLA, CD10)	Hs.307734	1.91	0.00
HSI028G12	*PDGFRA*	Platelet-derived growth factor receptor, alpha	Hs.74615	1.89	0.00
HSI060C05	*FN1*	Fibronectin 1	Hs.203717	1.89	0.00
HSI045G12	*CXCL12*	Chemokine ligand 12 (stromal cell-derived factor 1)	Hs.522891	1.88	0.00

Shown are the first 40 upregulated genes from a paired SAM analysis, which identified 1,365 genes to be upregulated by aromatase inhibitor (AI) treatment below a local false discovery rate of 1% (LFDR). The genes are ranked according to their fold change.

**Figure 3:**
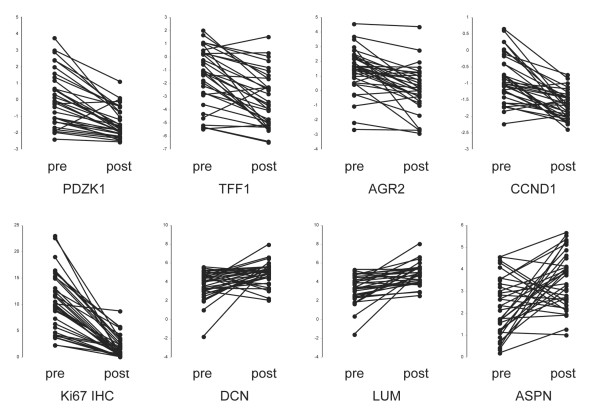
Expression changes in key index genes in response to AI treatment. Individual log ratio measurements are plotted and joined with a line in each of the paired biopsies. Individual results are shown for the downregulated genes *PDZK1*, *TFF1*, *AGR2 *and *CCND1*, and the upregulated genes *DCN*, *LUM *and *ASPN*. The percentage decrease in Ki67 immunohistochemistry (IHC) is shown in the bottom left panel for comparison.

The change in expression in some of these key index genes in individual patients is shown in Figure 3; the change in Ki67 immunohistochemistry is also shown for comparison. The majority of tumours exhibit large changes in expression of these genes. However, changes in the expression of individual estrogen-responsive genes did not clearly identify tumours with a poor antiproliferative response. Different subsets of tumours exhibited the largest or smallest responses in expression changes for each different gene.

**Figure 4:**
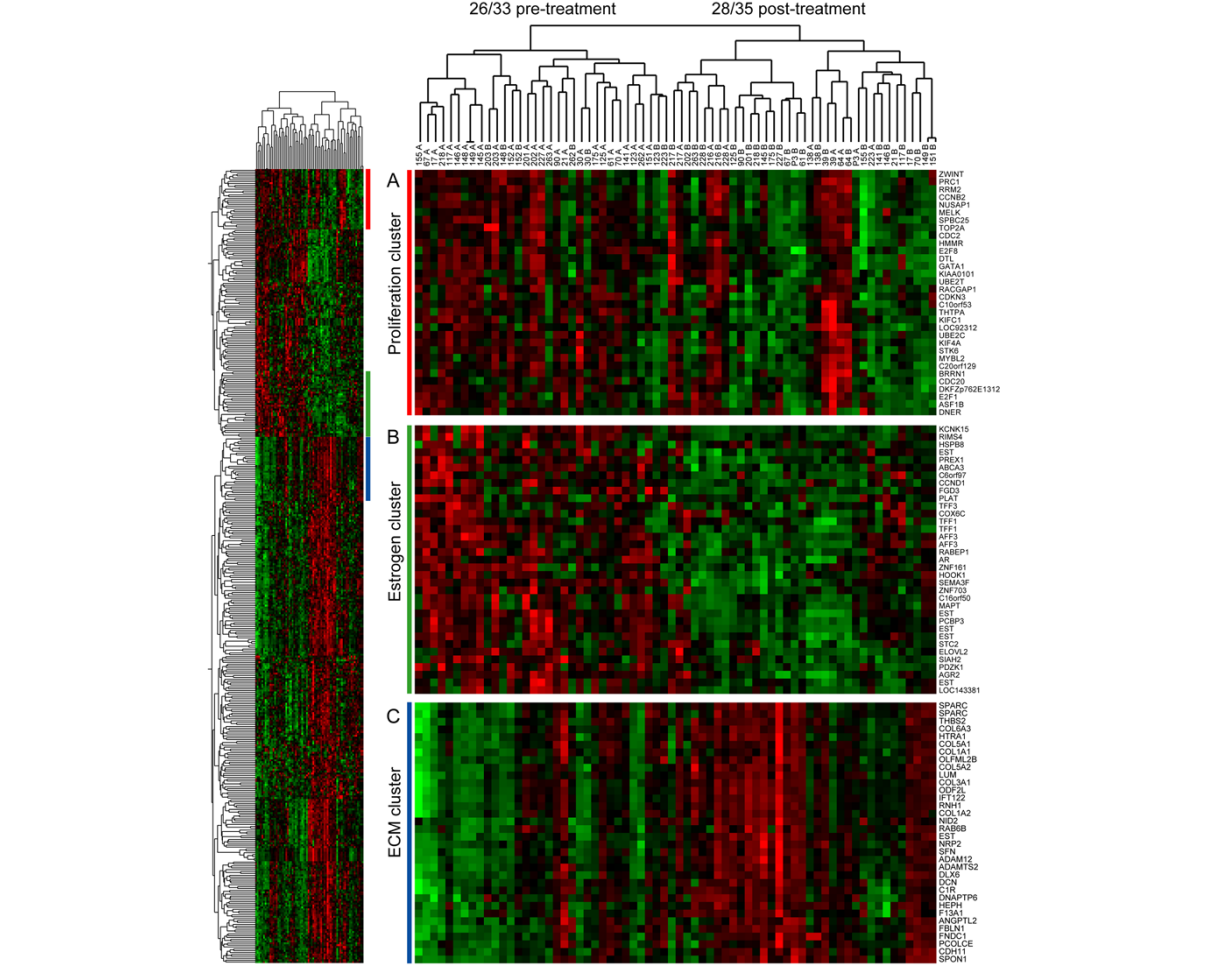
Supervised clustering of pre and post treatment biopsies. The 421 genes that best distinguished pretreatment and post-treatment biopsies were used to cluster the samples in the heatmap shown on the left. Three clusters of genes are shown in greater detail on the right: **(a) **a proliferation cluster representing genes associated with proliferation and cell cycle progression, **(b) **an estrogen cluster of known highly estrogen-responsive genes and **(c) **an extracellular matrix (ECM) cluster of genes known to be involved in ECM remodelling.

To map the gross phenotypic changes of the tumours in response to AI treatment relative to their initial states, we selected a core set of 421 genes that distinguished pretreatment from post-treatment biopies (see Materials and methods, above). These were used to produce the heat map shown in Figure 4 and separated the biopsies into predominantly pretreatment and post-treatment arms. Three of the four HER2 amplified cases had pretreatment profiles that segregated in the post-treatment arm (216, 228, and 64). The fourth (203) was the only case that expressed high levels of both *ESR1 *and *ERBB2*. Seven of the eight pretreatment biopsies that were incorrectly grouped included seven of the 10 biopsies with the lowest pretreatment expression of *ESR1 *(217, 216, 228, 138, 39, 64, and P3). Data files for Java Treeview are provided as supplementary information (Additional files 11, 12, 13, 14).

**Figure 5:**
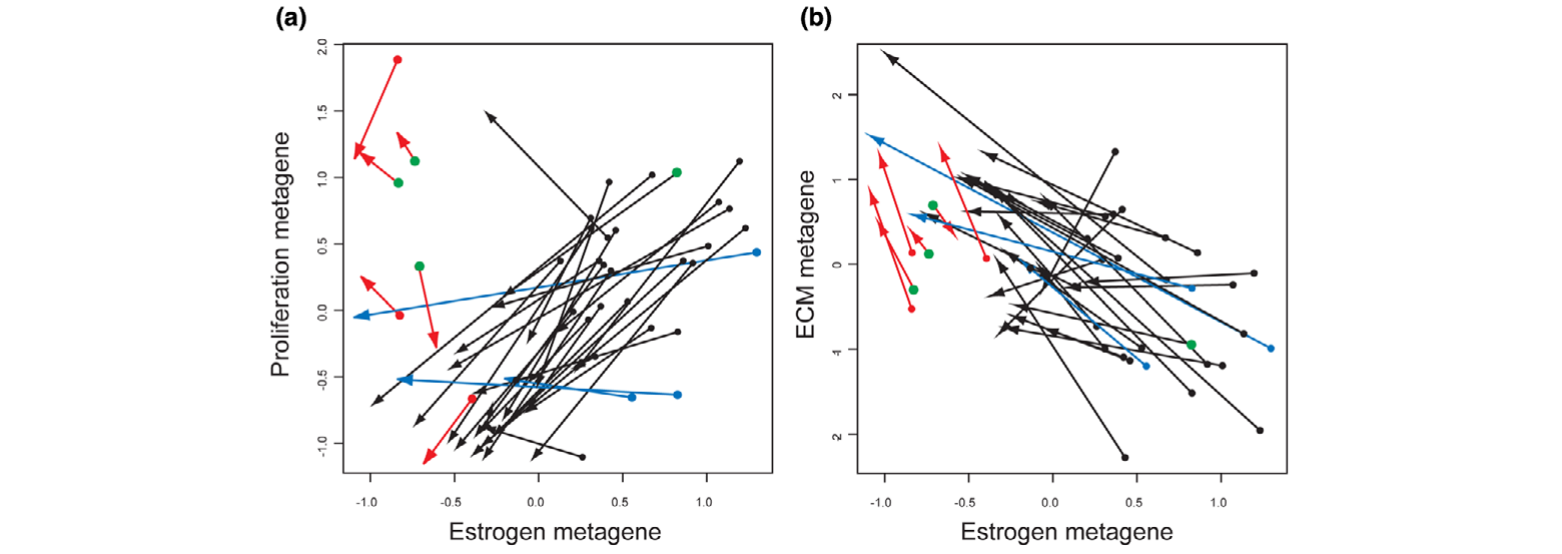
Vector diagrams of metagenes representing estrogen response, proliferation and ECM remodelling. Metagene values derived from the mean values of all the genes in each of the clusters in Figure 4 are plotted and connected with a line from dots (pretreatment values) to arrowheads (post-treatment values). Estrogen metagene values are compared with the **(a) **proliferation metagene and **(b) **the extracellular matrix (ECM). The six biopsies with the lowest pretreatment estrogen metagene are coloured in red. The four biopsies with HER2 amplification and high *ERBB2 *expression are shown with green dots, and samples with the lowest responses in the proliferation metagene are highlighted in blue.

Three clusters in this supervised analysis clearly represented distinct pathway related phenotypes based upon the ontology of the genes they contain (Figure 4). Genes in the 'proliferation cluster' exhibited a highly significant overlap with a previously characterized breast cancer proliferation signature [16]. We labelled a cluster containing many genes known to be classically estrogen responsive in breast cancer as an 'estrogen cluster' and one including collagens and other genes involved in extracellular matrix (ECM) deposition as an 'ECM cluster'. Figure 5a shows the combined effect of treatment on the estrogen and proliferation metagenes (mean of each cluster's M values) as a vector diagram in which the pretreatment and post-treatment samples are joined by an arrow. Tumours with extremely low baseline levels of estrogen-dependent gene expression and HER2 amplified tumours exhibit very little change in either cluster (for example, 39, 138 red arrows and 218, 216, 64, green dots, respectively). Perhaps most importantly, this analysis identified a number of cases that had major reductions in expression of the estrogen metagene with minimal impact on the proliferation metagene (for example, 145, 262, 263, blue arrows). Figure 5b shows the interaction of the estrogen metagene and the ECM metagene. The ECM metagene is clearly upregulated in the majority of biopsies irrespective of pretreatment levels of *ESR1 *and estrogen metagene values (red arrows). The proliferation metagene exhibited the highest positive correlation (*r *= 0.51, *P *= 0.000029) with the change in Ki67 immunohistochemistry of any of the nine metagenes (for example, estrogen metagene: *r *= 0.31, *P *= 0.102).

Array profiling also identified sets of genes that were both positively and negatively correlated with ER in these biopsies. The intersection of genes associated with ER and those identified as estrogen responsive indicated that only 10% of the genes most highly correlated with high *ESR1 *expression were downregulated by estrogen deprivation *in vivo*. A complete list of genes whose expression correlates with *ESR1 *with a Pearson correlation of greater or less than 0.5 is given in Additional file 5.

## Discussion

Anastrozole and letrozole are highly specific and efficient inhibitors of the aromatase enzyme, leading to profound estrogen deprivation in postmenopausal women [17]. These agents are also the most effective treatment for breast cancer in postmenopausal patients and have become the standard of care over recent years [2]. Here, we have used gene expression profiling by microarray to identify the longitudinal differences in gene expression between matched pretreatment and post-treatment biopsies of tumours from patients treated with AIs. The data generated in this study are biologically relevant in terms of identifying genes that respond to estrogen withdrawal in primary breast tumours *in vivo*, and are clinically relevant in identifying genes or groups of genes that may be used to understand and predict the response of patients to AI treatment. Although many reports have examined estrogen-regulated gene expression in breast cancer cells and model systems, generating a comprehensive genome-wide catalogue of estrogen-responsive genes [18], there are as yet few reported studies in which an AI was used as a biological probe of estrogen-dependent expression profiles in human breast carcinomas *in vivo *[19,20]. The number of patients included in our study was too small for confidence in matters of detail, but important broad messages may be developed.

Several studies have been reported over the past few years utilizing expression profiling of breast tumours, which demonstrated that the expression of ER by breast carcinomas is a consistently dominant feature in their transcriptional profile [13,21,22]. Although these studies have identified many hundreds of genes that are significantly associated with ER expression, it is has not been clear which of these genes are directly responsible for estrogen responses in tumour cells. The present study indicates that only a small proportion of the genes correlating with ER status are estrogen-responsive *in vivo*.

In the present study we included only ER-positive tumours (plus three tumours with Allred scores of 2, conventionally considered ER negative) [10]. Correlations between gene expression and ER in the current dataset were therefore made in relation to degree of ER expression rather than to ER positivity or negativity. Nonetheless, we observed strong correlations between ER and many genes that have previously been shown to be strongly associated with ER positivity, including *GATA3*, *FOXA1*, *AGR2*, *AR*, and *STC2*, in microarray profiling studies of mixed ER-positive and ER-negative tumours [21-23]. The present study indicates that only a small proportion of the genes correlating with ER status are estrogen responsive *in vivo*.

The GIDE may be a useful approach to characterizing the overall biological reactivity of a tumour to and dependence on estrogen. The data indicate that there is a continuum of such dependence, with 3,304 genes changing in one tumour by more than twofold over the 2-week treatment period, whereas only 105 changed in another. These data recapitulate the continuum of change shown by Ki67 immunohistochemistry, which indicates that almost all ER-positive tumours exhibit an antiproliferative response to estrogen deprivation, although this is highly variable between patients. The data from the GIDE similarly suggest that few ER-positive tumours are completely nonresponsive to estrogen deprivation. There was only a modestly significant relationship between the GIDE and the pretreatment level of ER based on immunohistochemistry; the current data suggest that high ER expression may be necessary for a tumour to be highly responsive (high GIDE) but that some tumours with a high ER have only a moderate or poor biological response.

The GIDE may be a useful end-point for investigating the mechanisms of resistance to hormonal therapy. One putative mechanism is through over-expression of growth factor receptors such as HER2. Although HER2 was associated with a low GIDE, in all but one case these tumours also had low ER, as has previously been observed [24]. PgR positivity has generally been regarded as indicative of an intact ER mechanism. An association with a higher GIDE might have been anticipated, and although there was a trend toward a positive association with higher PgR expression, this was not significant, possibly because of the limited numbers of samples. Although the GIDE would benefit from a proven association with clinical outcome, we recently showed that 2-week change in Ki67 was predictive of long-term outcome after treatment with endocrine agents in the adjuvant setting [6,25,26], and in this study the GIDE was found to be significantly associated with change in Ki67. The profound changes in transcriptional profiles found in some but not all tumours in this study suggest that it is possible that predicting clinical response to an AI by transcriptional profiling may, as with Ki67, be more precise when conducted in tumours shortly after starting treatment.

There have been many reports of the transcriptional profiling of estrogen responses in breast cancer cell lines *in vitro*, including those in MCF-7 [8,9], T47D [27] and ZR75.1 [28] breast cancer cell lines and their derivatives [29,30], as well as those using model systems in experimental animals [31,32]. These studies identified many hundreds of genes that were upregulated and downregulated by estrogen treatments. Computational and experimental attempts have also been made to integrate these data and catalogue all of the estrogen-responsive genes and estrogen response elements in the genome [18,33]. Many of the genes upregulated by estrogen *in vitro *were downregulated by AI treatment in our study including the majority of classically estrogen-responsive genes (*TFF1*, *TFF3*, *CYP2B6*, *PDZK1*, and *AGR2*). *TFF1 *(pS2) is one of the best characterized estrogen-responsive genes in breast cancer [8,34-36]. *CYP2B6 *is dramatically upregulated by estrogen in ZR75.1 cells, although it is not expressed in MCF-7 cells [37]. *PDZK1 *has consistently been identified as one of the genes most highly upregulated by oestradiol in MCF-7 cells [8,9]. *AGR2 *is another classically estrogen-responsive gene that is expressed in both cell lines and ER-positive breast tumours [38] and that has been associated with a poor response to hormonal therapy [39]. One of the genes that we found to be significantly downregulated by AIs was that encoding aromatase itself (*CYP19A1*). This finding supports earlier evidence of a positive autocrine feedback loop [40].

In contrast to the genes downregulated by AI treatment, the upregulated genes are not represented by those that are directly downregulated by estrogen in cell lines *in vitro*. Gene Ontology analysis of the upregulated genes identified pathways involved with the regulation of the actin cytoskeleton, cytokine-receptor interactions and focal adhesion to be more commonly associated with the functions of stromal components than of epithelial cells (*VIM*, *CTGF*, *FN1*, and *SPARC*). The genes most highly upregulated by AI treatment include several members of the of the small leucine-rich proteoglycan family (*LUM*, *ASPN*, and *DCN*), which regulate matrix remodelling. Lumican is not expressed in cancer cells in breast cancers but in fibroblasts, and it is associated with high tumour grade, low ER levels and young age [41]. Decorin is preferentially expressed in stromal areas in proliferating endometrium, is directly upregulated by estrogens in stromal endometrial cells *in vitro *[42] and is upregulated in mouse uterus by estrogen treatment [43]. Asporin is closely related to biglycan, which has been shown to be downregulated by estrogen in the stroma of normal human breast tissue in a mouse xenograft model [32]. The effect of this stromal signature on patient survival is unclear but reduced small leucine-rich proteoglycan family expression has been observed in poor prognosis ER-negative breast cancer [44].

There are several possible mechanisms for this upregulation of a stromal signature clearing response to AI treatment. It was notable that the genes representing this stromal signature were upregulated independent of high level ER-α expression in tumour cells. It is possible that upregulation may result from an interaction with stromally expressed ER-β [45]. For example, *CD36 *has been shown to be directly upregulated by estrogen via ER-β [46], and both lumican and *PDGFRA *were induced by the selective ER modulator raloxifene in U2OS cells transfected with ER-β [30]. Among the other genes upregulated by AI treatment in the present study are genes representative of the normal profiles of luminal and myoepithelial phenotypes [47,48] that are not driven by high-level ER over-expression, including *RARRES1*, *MME*, *TCF4*, *SFN*, and *CAV1*. This represents a joint upregulation in post-treatment biopsies of a basal/stromal phenotype, which has also been shown in estrogen treatments of normal human breast tissue in xenograft studies [32].

Taken together, these findings highlight the fact that studies identifying estrogen-responsive genes in cell lines do not take into account the diversity of responsiveness, composition and genetic backgrounds seen in primary ER-positive breast tumours. Although many of the gene changes are likely to be directly transcriptionally regulated by estrogen, it is also likely that the majority are a secondary consequence of estrogen deprivation and the resulting inhibition of breast tumour proliferation by AI treatment. Recently, Oh and coworkers [38] have attempted to integrate data on estrogen responsiveness in MCF-7 cells *in vitro *with gene expression and clinical outcome data from 65 ER-positive and/or PgR-positive breast cancer patients to predict outcome for hormone responsive breast cancer. The study used only the 383 genes that were upregulated by estrogen treatment in this single cell line but, a very high dose (1 μmol/l) of oestradiol was used and dosage differences have been suggested to compromise comparisons of transcriptional signatures [49]. The identification of a comprehensive profile of estrogen-responsive genes in tumours deprived of estrogen *in vivo *may be expected to provide a much better basis on which to classify the estrogen response of breast tumours than *in vitro *studies.

Robust gene selection methods were used to identify genes that together best separated pretreatment from post-treatment samples. Cluster analysis using these genes identified groups associated with proliferation and estrogen responses. The 32 genes that constitute the 'proliferation cluster' contain 17 of those reported by Dai and coworkers [16] as a proliferation signature containing critical genes predicting the long-term clinical outcome of patients with ER-positive breast tumours. To summarize both the 'estrogen cluster' and the 'proliferation cluster', we used metagene values to depict the relative changes in tumour phenotype in response to AI treatment. In most tumours there was a coordinated decrease in both of these clusters, but we observed that in some tumours these facets of phenotype change were uncoupled. A better understanding of the mechanisms that lead to a poor antiproliferative response in the presence of a good response in the 'estrogen cluster' of genes is likely to provide a guide to additional treatments for ER-positive breast cancer and may be possible with an extension of this study to larger numbers of tumours.

## Conclusion

In summary, short-term estrogen deprivation with AIs leads to profound changes in transcriptional profiles. Although many of the genes were previously described in cell culture studies as responsive to estrogen stimulation, many additional estrogen-responsive genes were identified that responded to estrogen deprivation *in vivo*, particularly those that are repressed by estrogen. The study revealed complex changes in estrogen-responsive pathways, proliferation and matrix remodelling, which cannot be simply summarized by the ER status of the tumours or completely recapitulated in cell line studies. The global changes in gene expression can be integrated into a GIDE that we found to be associated with previously established correlates with clinical outcome. Studies of this type, linked with clinical outcome, should enable the key genes that underpin clinical response/benefit to be established and may be expected to reveal the molecular features of tumours responsible for sensitivity and resistance to estrogen deprivation.

## Abbreviations

AI = aromatase inhibitor; ER = estrogen receptor; ECM = extracellular matrix; GIDE = Global Index of Dependence on Estrogen; PCR = polymerase chain reaction; PgR = progesterone receptor.

## Competing interests

MD serves on advisory boards and receives grant income from Astrazeneca and Novartis in relation to AIs. The other authors declare that they have no competing interests.

## Authors' contributions

AM and AU carried out the microarray profiling, assisted in data analysis and drafted the manuscript. AM and KF produced the microarrays used in this study, TD carried out the Bioinformatic and statistical analyses. SD and AL carried out the RT-PCR validations. OY and SW carried out the immunohistochemistry. JMD, WM, AA, DE and MD participated in the design and conception of the study, and reviewed analyses of the data. MD, JMD and WM designed the study, and AA supported the laboratory work. All authors read and approved the final manuscript.

## Supplementary Material

Additional file 6 Annotation of the Breakthrough 17K microarray Click here for file

Additional file 1 Primer sequences used in RT-PCR Click here for file

Additional file 2 A summary of relevant patient information Click here for file

Additional file 7 Java Treeview data for figure 1 Click here for file

Additional file 8 Java Treeview data for figure 1 Click here for file

Additional file 9 Java Treeview data for figure 1 Click here for file

Additional file 10 Complete annotation of the genes in the 2418 heatmap Click here for file

Additional file 3 A table of individual results used to plot GIDE correlations in figure 2 Click here for file

Additional file 4 An excel sheet listing all genes significantly up- and downregulated by AI treatment Click here for file

Additional file 11 Java Treeview data for figure 4 Click here for file

Additional file 12 Java Treeview data for figure 4 Click here for file

Additional file 13 Java Treeview data for figure 4 Click here for file

Additional file 14 Complete annotation of the genes in the 421 heatmap Click here for file

Additional file 5 An excel sheet listing all genes positively and negatively correlated with ESR1 expression Click here for file
